# Supplementary material for: C9orf72-ALS human iPSC microglia are pro-inflammatory and toxic to co-cultured motor neurons via MMP9
Source: Nat Commun. 2023 Sep 22;14:5898. doi: 10.1038/s41467-023-41603-0 (PMC10517114; doi:10.1038/s41467-023-41603-0)
Supplement: Supplementary file 1 — Supplementary Information [file 41467_2023_41603_MOESM1_ESM.pdf]

SUPPLEMENTARY MATERIAL

C9orf72-ALS human iPSC microglia are pro-inflammatory and toxic to co-cultured motor neurons via MMP9

Björn F. Vahsen[1,2], Sumedha Nalluru[1], Georgia R. Morgan[1], Lucy Farrimond[1,2], Emily Carroll[1,2], Yinyan Xu[1,2,3], Kaitlyn M. L. Cramb[2,4], Benazir Amein[1], Jakub Scaber[1,2], Antigoni Katsikoudi[2,5], Ana Candalija[1], Mireia Carcolé[6], Ruxandra Dafinca[1,2], Adrian M. Isaacs[6], Richard Wade-Martins[2,4], Elizabeth Gray[1], Martin R. Turner[1], Sally A. Cowley[7,*], Kevin Talbot[1,2,*]

*** to whom correspondence should be addressed**

Author affiliations:

1 Oxford Motor Neuron Disease Centre, Nuffield Department of Clinical Neurosciences, University of Oxford, John Radcliffe Hospital, Oxford OX3 9DU, UK

2 Kavli Institute for Nanoscience Discovery, University of Oxford, Dorothy Crowfoot Hodgkin Building, Oxford OX1 3QU, UK

3 Chinese Academy of Medical Sciences (CAMS), CAMS Oxford Institute (COI), Nuffield Department of Medicine, University of Oxford, Oxford OX3 7FZ, UK

4 Oxford Parkinson's Disease Centre, Department of Physiology, Anatomy and Genetics, University of Oxford, Dorothy Crowfoot Hodgkin Building, Oxford OX1 3QX, UK

5 Molecular Neurodegeneration Research Group, Nuffield Department of Clinical Neurosciences, University of Oxford, Dorothy Crowfoot Hodgkin Building, Oxford OX1 3QU, UK

6 UK Dementia Research Institute at UCL and Department of Neurodegenerative Disease, UCL Queen Square Institute of Neurology, London, WCIN 3BG, UK

7 James and Lillian Martin Centre for Stem Cell Research, Sir William Dunn School of Pathology, University of Oxford, Oxford OX1 3RE, UK

Supplementary Tables:

Supplementary Table 1: Human induced pluripotent stem cell lines used in this study. PMID: PubMed ID. HC-2b was used to generate MNs; microglia were derived from all other lines.

iPSC line	abbreviation	sex	age	mutation	PMID
OX3-06	HC-1	male	49	-	29604226
SFC856-03-04	HC-2a	female	78	-	28827786
SFC840-03-03	HC-2b	female	67	-	26905200
SFC841-03-01	HC-3	male	36	-	27097283
C9-01-07	C9-1	male	72	*C9orf72* (~970 G_4C_2 repeats)	32330447
C9-02-02	C9-2	female	58	*C9orf72* (~1000 G_4C_2 repeats)	27097283
C9-04-12	C9-3	male	39	*C9orf72* (>500 G_4C_2 repeats)	32330447
59-2	ΔC9-2	female	58	isogenic control (2 G_4C_2 repeats)	32504093

Supplementary Table 2: Primary antibodies used for immunofluorescent staining in this study.

target	species	dilution	catalog number	company
ChAT	goat	1:100	ab144P	Sigma-Aldrich
cleaved caspase 3	rabbit	1:400	9661S	Cell Signaling
DPP4	rabbit	1:200	67138 (Clone: D6D8K)	Cell Signaling
IBA1	rabbit	1:500	019-19741	FUJIFILM Wako
IBA1	goat	1:500	ab5076	Abcam
synaptophysin	guineapig	1:500	101004	Synaptic Systems
TDP-43	mouse	1:500	60019-2-1g (Clone: 6H6E12)	Proteintech
TMEM119	rabbit	1:100	ab185337	Abcam
TUJ1	mouse	1:1000	801201 (Clone: TUJ1)	Biolegend

Supplementary Table 3: Primers used for RT-qPCR in this study.

Target gene	Forward primer sequence	Reverse primer sequence
C9orf72 (all)	CCCACTTCATAGAGTGTGTGTTG	TTCCATTCTCTCTGTGCCTTC
C9orf72 V1 (short)	GAAATCACACAGTGTTCCTGAAGAA	ATCTGCTTCATCCAGCTTTTATGA
C9orf72 V2+3 (long)	CATGGCTCAGGATACGATCA	GGAAGGCTTTCACTAGAGTGTCTC
CXCL10	GTGGCATTCAAGGAGTACCTC	TGATGGCCTTCGATTCTGGATT
MMP9	GGGACGCAGACATCGTCATC	TCGTCATCGTCGAAATGGGC
P2RY12	AAGAGCACTCAAGACTTTAC	GGGTTTGAATGTATCCAGTAAG
TBP	GAGAGTTCTGGGATTGTACCG	ATCCTCATGATTACCGCAGC

TMEM119	AGTCCTGTACGCCAAGGAAC	GCAGCAACAGAAGGATGAGG

Supplementary Table 4: Primary antibodies used for Western blot in this study.

target	species	dilution	catalog number	company
b-actin	mouse	1:5000	AM4302 (Clone: AC-15)	ThermoFisher
C9orf72	mouse	1:500	GTX634482 (Clone: GT1553)	GeneTex
C9orf72	mouse	1:1000	GTX632041 ((Clone: GT779)	GeneTex
ChAT	goat	1:1000	ab144P	Sigma-Aldrich
GAPDH	mouse	1:1000	Nb300221 (Clone: 1D4)	Novus Biologicals
IBA1	rabbit	1:1000	019-19741	FUJIFILM Wako
MMP9	rabbit	1:1000	ab76003 (Clone: EP1254)	Abcam

Supplementary Table 5: Cytokine release into the culture supernatant measured using the Proteome Profiler Human XL Cytokine Array Kit on pooled supernatant samples from unstimulated and LPS-treated (100 ng/mL, 48 h) C9orf72-2 (C9-2) and isogenic control (IC) microglia in monoculture (pMGL) (pooled samples from n=1 line per condition with n=3 differentiations each). Data represent the mean of two technical replicates per condition. Values correspond to the signal intensity value expressed as arbitrary units. Chemokines/cytokines of particular interest and plotted in Supp. Fig. 5 are highlighted in bold.

released factor	IC -LPS	IC +LPS	C9-2 -LPS	C9-2 +LPS
Adiponectin	750.7241	467.6934	270.3868	563.1321
Apolipoprotein A-I	4561.724	3674.443	2447.887	3074.632
Angiogenin	5934.224	5141.193	2150.887	3614.132
Angiopoietin-1	407.7241	575.6934	245.3868	331.1321
Angiopoietin-2	1576.724	1919.693	827.3868	1881.132
BAFF	859.2241	861.6934	287.3868	786.1321
BDNF	7607.724	5765.193	3975.387	8191.132
C5/C5a	461.2241	651.6934	150.3868	1085.632
CD14	3619.224	3824.443	2428.387	3882.132
CD30	157.7241	356.1934	68.38679	61.63208
CD40 ligand	569.2241	569.4434	0	459.6321
Chitinase 3-like 1	23569.22	17324.19	15554.14	18127.63
Complement Factor D	2150.224	1786.193	438.8868	1342.382
C-Reactive Protein	869.7241	981.6934	334.3868	1240.132
Cripto-1	166.7241	457.1934	240.3868	360.1321
Cystatin C	875.7241	450.1934	197.3868	563.1321
Dkk-1	7836.224	5875.693	4757.887	3987.132
DPPIV	11540.22	12829.69	9391.887	18334.13
EGF	293.2241	408.1934	0	0
Emmprin	1326.224	1263.693	828.8868	1671.132
ENA-78	19426.22	18231.69	7041.387	19742.63
Endoglin	1980.724	1521.193	1011.387	1359.632
Fas Ligand	456.7241	635.6934	303.8868	476.1321
FGF basic	553.2241	841.6934	266.8868	1168.632
FGF-7	200.2241	370.6934	18.88679	398.6321
FGF-19	3574.724	3664.693	1641.887	3876.132
Flt-3 Ligand	554.2241	516.6934	258.3868	213.1321
G-CSF	601.7241	1667.693	146.3868	950.6321
**GDF-15**	**733.2241**	**864.1934**	**3364.887**	**4368.632**
GM-CSF	725.2241	1503.693	350.8868	1212.132
GROα	9068.474	8211.693	5615.887	10101.13
Growth Hormone	165.2241	116.1934	0	6.632075
HGF	1022.224	1235.693	487.3868	1213.632

ICAM-1	2405.224	1816.693	623.6368	3203.632
IFN-γ	869.2241	792.6934	735.8868	793.1321
IGFBP-2	12969.97	12778.19	8963.887	11425.13
IGFBP-3	4613.224	3095.693	1763.887	2838.132
IL-1α	543.2241	404.1934	221.3868	640.6321
IL-1β	447.7241	337.1934	303.8868	462.6321
IL-1ra	1009.224	2891.693	970.8868	3724.382
IL-2	640.7241	576.6934	426.3868	983.1321
IL-3	87.22406	181.1934	120.3868	126.1321
IL-4	1069.724	644.6934	198.8868	1517.132
IL-5	13.22406	0	0	22.63208
IL-6	10093.72	16804.69	2480.387	17699.13
IL-8	26956.22	18741.69	18904.89	20429.13
IL-10	1911.224	3257.693	754.8868	3558.132
IL-11	1590.224	2075.693	919.3868	1478.632
IL-12 p70	497.2241	509.1934	64.88679	693.6321
IL-13	155.7241	296.1934	133.8868	135.1321
IL-15	147.2241	91.6934	103.8868	326.1321
IL-16	160.2241	200.1934	64.88679	499.6321
IL-17A	4089.724	5942.193	4234.887	6589.132
IL-18 Bpa	210.7241	361.1934	611.8868	722.1321
IL-19	480.7241	261.1934	57.88679	257.6321
IL-22	1924.724	1826.693	787.3868	1918.632
IL-23	97.22406	877.6934	194.3868	76.63208
IL-24	247.2241	604.9434	0	0
IL-27	1326.224	1933.693	791.3868	2053.132
IL-31	357.7241	8.693396	0	292.6321
IL-32	228.2241	280.6934	56.88679	418.6321
IL-33	109.2241	214.1934	122.3868	98.63208
IL-34	663.2241	164.6934	535.3868	554.6321
**IP-10**	**3235.724**	**26647.69**	**1480.387**	**8244.632**
I-TAC	287.2241	457.1934	178.3868	375.1321
Kallikrein 3	1028.224	1109.693	2354.887	2733.632
Leptin	466.2241	120.6934	20.88679	348.1321
LIF	311.7241	732.1934	0	618.6321
Lipocalin-2	950.2241	896.6934	470.8868	921.1321
MCP-1	14549.72	14633.19	12557.39	13890.13
MCP-3	785.7241	16825.69	496.8868	14583.13
M-CSF	776.7241	1140.693	600.8868	1251.632
MIF	5031.724	4591.193	3925.387	4684.132
MIG	314.7241	535.6934	241.3868	437.6321
MIP-1α/MIP-1β	1124.724	9578.193	800.3868	13301.38
MIP-3α	1445.974	15403.69	888.8868	19439.13
MIP-3β	268.7241	0	579.3868	758.1321
**MMP-9**	**17489.72**	**21324.69**	**25124.39**	**31134.63**
Myeloperoxidase	780.2241	627.1934	149.8868	0
Osteopontin	14854.72	15208.69	10418.89	15807.13
PDGF-AA	862.7241	1376.193	485.3868	978.6321
PDGF-AB/BB	63.72406	59.6934	0	122.6321
Pentraxin 3	5895.724	9566.193	4990.387	6521.132
PF4	313.2241	269.1934	77.38679	0
RAGE	229.2241	373.6934	283.3868	243.6321
RANTES	340.7241	2265.193	325.8868	2448.632
RBP-4	485.7241	236.1934	396.3868	667.6321
Relaxin-2	257.7241	79.6934	94.88679	820.1321
Resistin	1847.224	13868.94	352.8868	5323.632
SDF-1α	4270.224	4503.193	3442.887	6421.132
Serpin E1	23322.72	17522.94	16795.89	19995.13
SHBG	58.22406	59.6934	0	757.6321
ST2	0	289.1934	83.88679	34.63208
TARC	35.72406	439.1934	199.8868	147.6321
TFF3	133.2241	303.1934	172.8868	0
TfR	632.7241	483.1934	241.3868	673.6321
TGF-α	114.2241	311.6934	144.3868	261.6321
Thrombospondin-1	2796.724	3121.193	2796.387	3330.132
TNF-α	564.2241	2116.693	406.3868	1856.632
uPAR	4604.724	5744.193	3291.887	9787.632
VEGF	318.7241	1077.693	179.8868	469.6321
Vitamin D BP	691.2241	1020.193	750.3868	667.6321
CD31	855.2241	1663.693	1233.387	1366.632
TIM-3	5288.224	8376.193	7749.387	8258.882
VCAM-1	2486.224	5206.193	2087.887	3399.632

Supplementary Figures and Figure Legends:

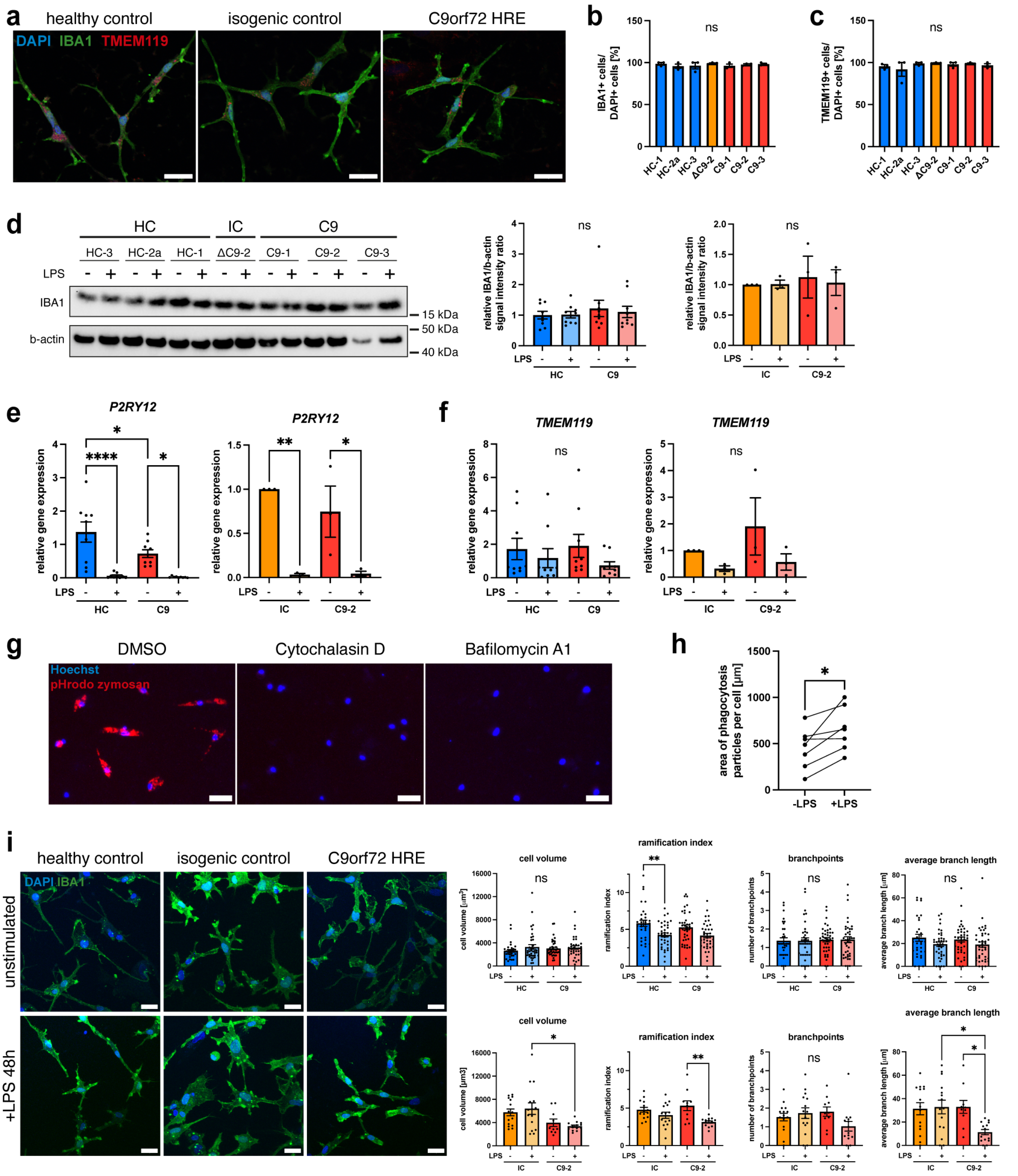

Supp. Fig. 1: Complementary assessment of microglial marker expression and cell morphology in C9orf72 mutant microglia. a) Exemplar images of healthy control (HC), isogenic (IC), and C9orf72 mutant (C9) microglia in monoculture (pMGL) showing branched microglial morphology and expression of the microglial markers IBA1 and TMEM119. Scale bars: 25 µm. **b/c)** Quantification of the percentage of cells expressing the microglial markers IBA1 (**b**) and TMEM119 (**c**) after differentiation of microglia from each line used in this study (n=3 differentiations per line). **d)** Left: Exemplar Western blot against the microglial marker IBA1 in unstimulated and LPS-treated (100 ng/mL, 48 h) HC, IC, and C9 pMGL. Right: Quantification showing equal IBA1 expression in the C9-HC pMGL (n=3 lines per condition,

n=3 differentiations each) and IC-C9-2 pMGL comparisons (n=1 line per condition, n=3 differentiations each) normalized to the housekeeping gene b-actin. **e/f)** RT-qPCR for *P2RY12* and *TMEM119* expression in unstimulated and LPS-treated pMGL normalized to the housekeeping gene *TBP* shows reduced *P2RY12* expression in C9 pMGL versus controls, while *TMEM119* expression is equal (n=3 lines per condition in the C9-HC pMGL comparison; n=1 line per condition in IC-C9-2 pMGL comparison; n=3 differentiations per line). **g)** Exemplar images showing phagocytic uptake of pHrodo zymosan particles in HC microglia, which is abolished by Cytochalasin D (phagocytosis blocker) and Bafilomycin A1 (blocker of lysosomal degradation). Scale bars: 50 µm. **h)** Quantification showing phagocytic uptake of pHrodo zymosan particles in microglia differentiated from all lines in this study. 48h pre-treatment with LPS significantly increases phagocytic uptake (n=7 lines per condition, n=1 differentiation per line). **i)** Left: Exemplar images of unstimulated and LPS-treated HC, IC, and C9 pMGL. Right: Quantification of different cell morphology parameters using 3DMorph shows no differences between C9 and HC pMGL (n=3 lines per condition, n=3 differentiations each). In the IC-C9-2 pMGL comparison, cell volume and average branch length are significantly reduced in LPS-stimulated C9-2 pMGL (n=1 line per condition, n=3 differentiations each). Datapoints are single cells. Scale bars: 25 µm. Single data points and means ± SEM. Two-tailed paired t-test (**h**). One-way ANOVA with Tukey's post-hoc test (**b-f, i**). Source data are provided as a Source Data file.

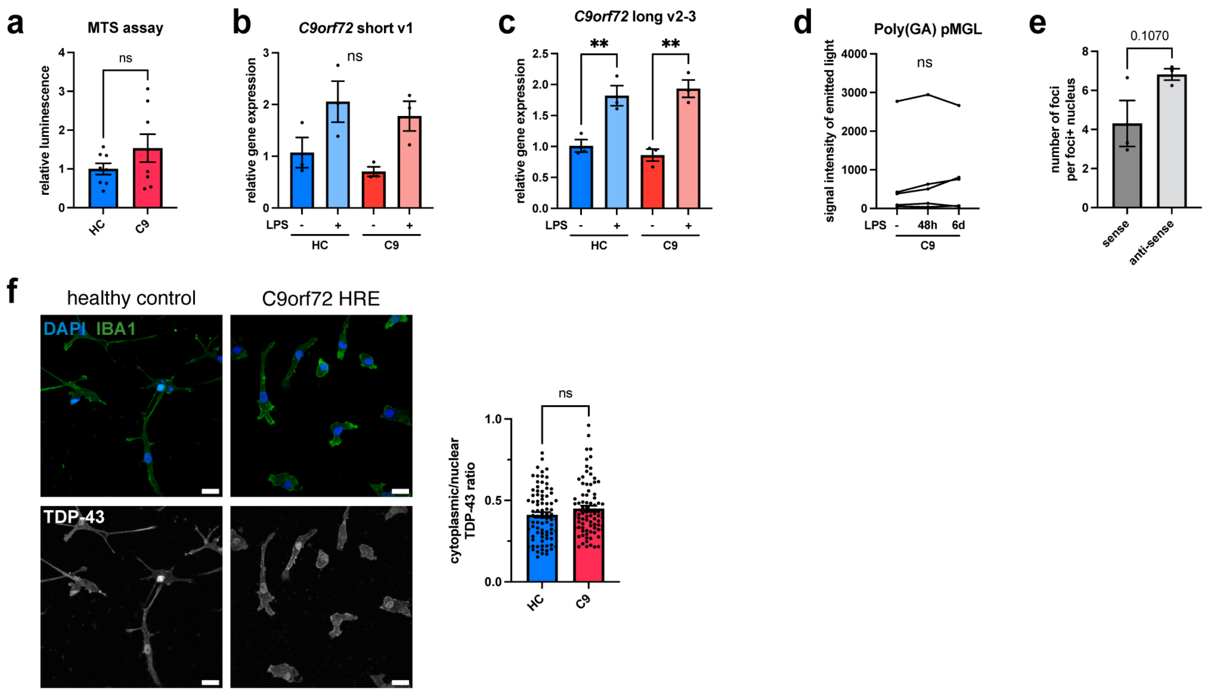

Supp. Fig. 2: Complementary assessment of *C9orf72* variants and pathological hallmarks associated with the HRE in *C9orf72* in C9orf72 mutant iPSC microglia. a) Quantification of relative cell viability using an MTS assay showing no significant difference between C9orf72 mutant (C9) and healthy control (HC) microglia in monoculture (pMGL) (n=2 lines per condition, n=4 differentiations each). **b)** RT-qPCR for variant 1 (v1, encoding the short isoform) and variants 2/3 (v2-3, encoding the long isoform) of *C9orf72* in unstimulated and LPS-stimulated pMGL normalized to the housekeeping gene *TBP*. Both are significantly upregulated after LPS treatment, but there is no significant difference between C9 and HC pMGL (n=3 lines per condition, n=1 differentiation per line). **d)** Analysis of Poly(GA) expression in pMGL after 48 h and 6 d of LPS treatment (100 ng/mL) by MSD ELISA shows no changes in response to treatment (n=3 lines per condition n=1-2 differentiations per line). **e)** Quantification of the number of RNA foci per foci-positive cell showing no significant difference between sense and anti-sense foci (n=3 line per condition, n=1 differentiation each). **f)** Left: Exemplar images showing TDP-43 distribution in unstimulated and LPS-stimulated HC and C9 pMGL. Right: Quantification showing no difference in the cytoplasmic/nuclear TDP-43 ratio comparing HC and C9 pMGL. Data points are individual cells from n=3 differentiations for n=3 lines per condition. Scale bars: 20 µm. Single data points and means ± SEM. Two-tailed unpaired t-test (**a/e/f**). One-way ANOVA with Tukey's post-hoc test (**b-d**). Source data are provided as a Source Data file.

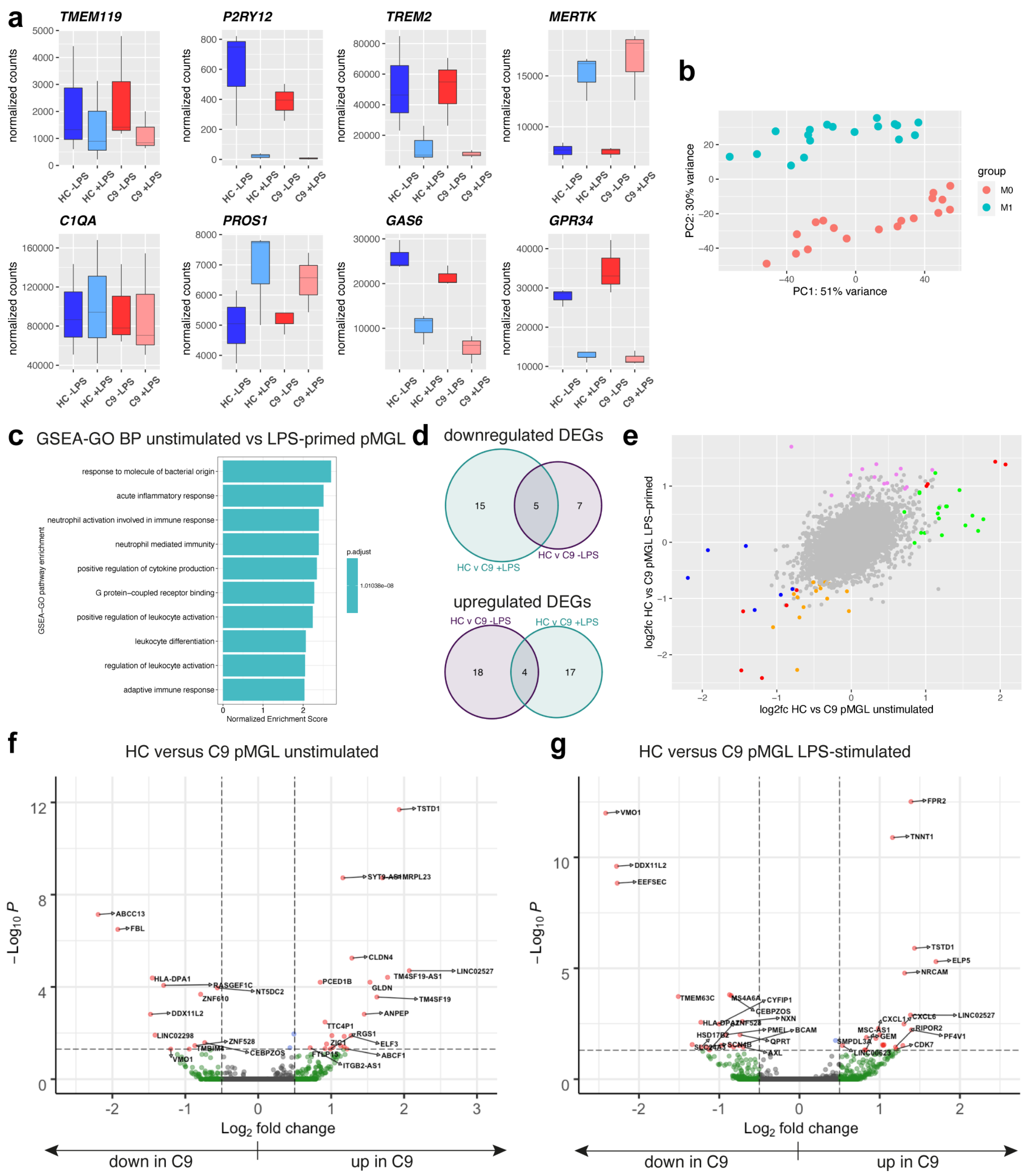

Supp. Fig. 3: Complementary analysis of RNA sequencing of C9orf72 mutant iPSC microglia. a) Box plots of normalized counts from RNA seq analysis confirming the expression of the microglial markers *TMEM119*, *P2RY12, TREM2, MERTK, C1QA, PROS1, GAS6,* and *GPR34* in unstimulated and LPS-treated (100 ng/mL, 48 h) healthy control (HC) and C9orf72 mutant (C9) microglia in monoculture (pMGL) (n=3 lines per condition). **b)** PCA plot based on the top 500 most variable genes with biggest variance in unstimulated versus LPS-treated pMGL (n=3 lines per condition and genotype, n=3 differentiations each). **c)** Bar plot confirming enrichment of immune cell activation pathways in LPS-treated pMGL. x-axis showing normalized enrichment score (NES) based on gene set enrichment analysis (GSEA) using the whole transcriptome ranked by log_2fc (n=3 lines per condition and genotype, n=3 differentiations each). The Benjamini-Hochberg corrected p-values for pathway enrichment for each term is indicated by the color of the bars. **d)** Venn diagrams showing overlap of

downregulated and upregulated DEGs in unstimulated and LPS-primed C9 versus HC pMGL (n=3 lines per condition). **e)** Scatter plot showing log_2fc in unstimulated and LPS-primed C9 versus HC pMGL (n=3 lines per condition, positive = enriched in C9 pMGL). DEGs are highlighted in different colors – red: DEGs overlapping between unstimulated and LPS-stimulated C9 pMGL compared with HC pMGL ; blue: DEGs downregulated in unstimulated C9 pMGL; green: DEGs upregulated in unstimulated C9 pMGL; orange: DEGs downregulated in LPS-stimulated C9 pMGL; violet: DEGs upregulated in LPS-stimulated C9 pMGL. **f/g)** Volcano plot showing DEGs in unstimulated (**f**) and LPS-stimulated (**g**) C9 pMGL vs HC (n=3 lines per condition).

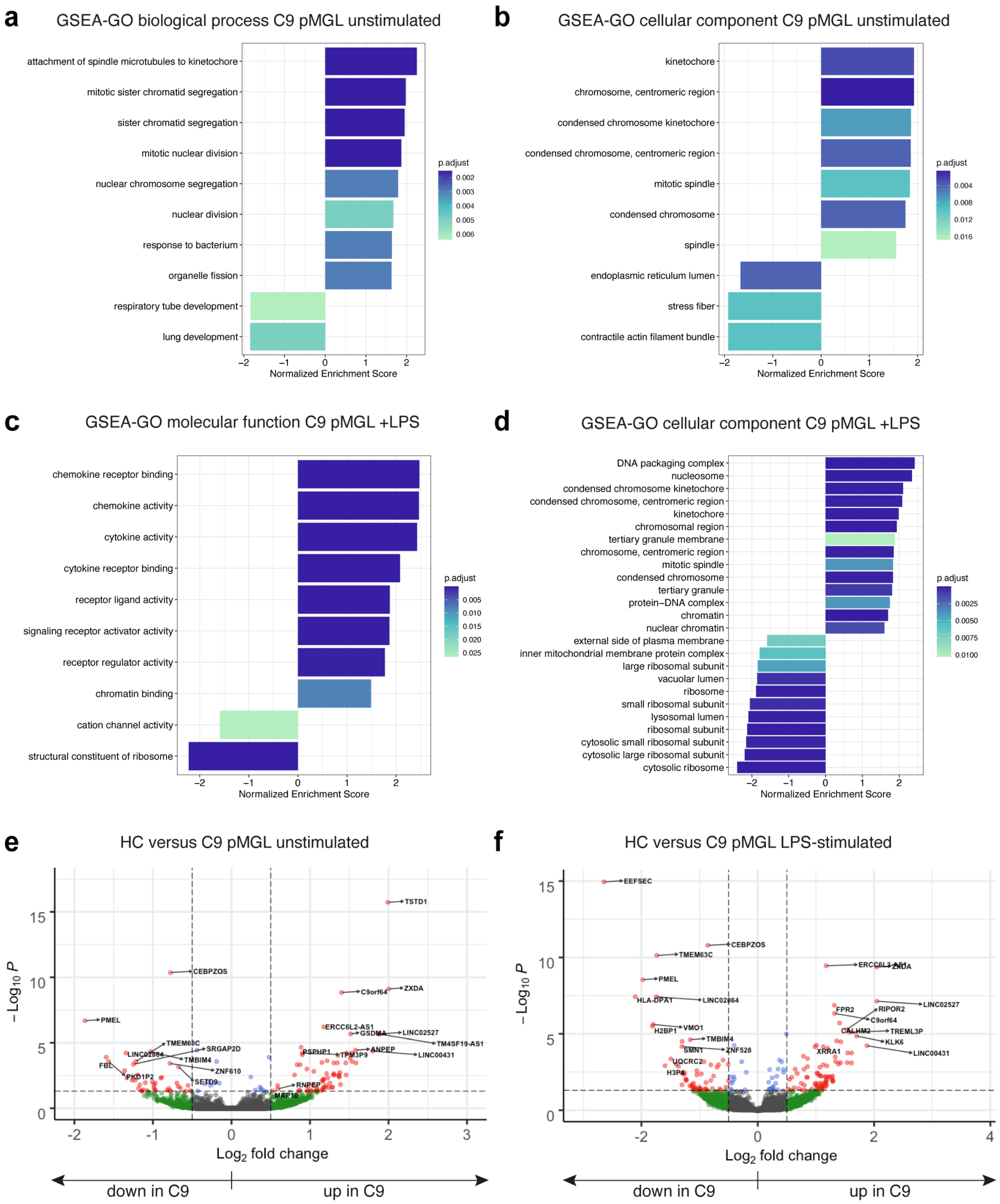

Supp. Fig. 4: Complementary analysis of RNA sequencing of C9orf72 mutant iPSC microglia. a/b) Bar plots showing top 10 significantly different GO biological process (**a**) and cellular component (**b**) terms for unstimulated C9 pMGL (n=3 lines per condition) versus HC pMGL based on gene set enrichment analysis (GSEA) using the whole transcriptome ranked by log_2fc. x-axis showing normalized enrichment score (NES). The Benjamini-Hochberg corrected p-values for pathway enrichment for each term is indicated by the color of the bars. **c/d)** Bar plots showing top 10 significantly different GO molecular function (**c**) and top 25 cellular component (**d**) terms for LPS-stimulated C9 pMGL (n=3 lines per condition) versus HC pMGL based on gene set enrichment analysis (GSEA) using the whole transcriptome ranked by log_2fc. x-axis showing normalized enrichment score (NES). The Benjamini-

Hochberg corrected p-values for pathway enrichment for each term is indicated by the color of the bars. **e/f)** Volcano plot showing DEGs in unstimulated (**e**) and LPS-stimulated (**f**) C9 vs HC pMGL with n=3 different differentiations for each line (n=3) used as different datapoints.

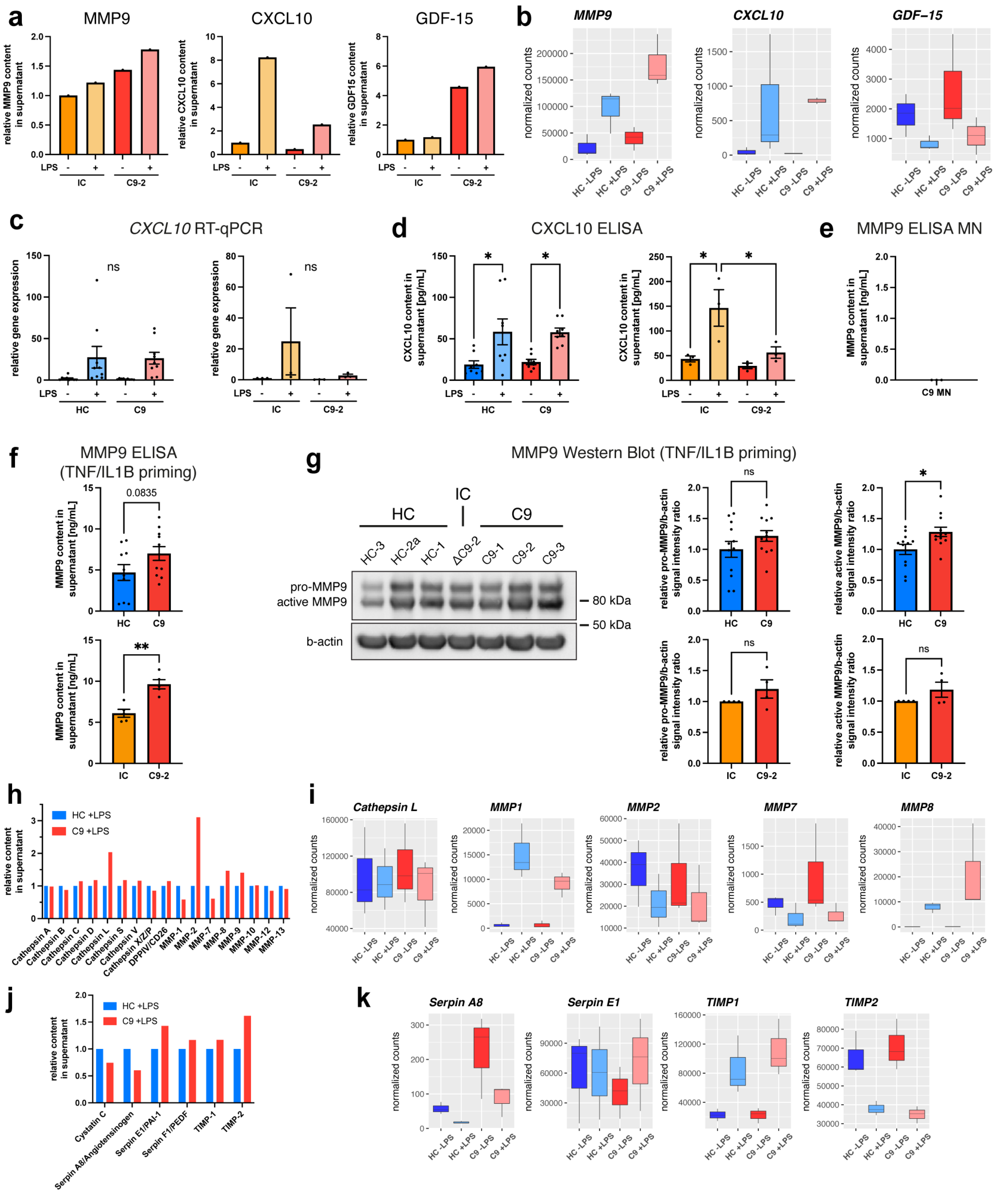

Supp. Fig. 5: Complementary analysis of the cytokine expression and supernatant profile in C9orf72 mutant iPSC microglia. a) Relative fold change in supernatant content for MMP9, CXCL10, and GDF-15 using a Cytokine Array on unstimulated and LPS-treated (100 ng/mL, 48 h) C9orf72-2 mutant (C9-2) and isogenic (IC) microglia in monoculture (pMGL) (pooled from n=3 differentiations each). Full list of chemokines/cytokines provided in Supplementary Table 5. **b)** Box plots of normalized counts from RNA seq analysis for *MMP9*, *CXCL10*, and *GDF-15* (n=3 lines per condition). **c)** RT-qPCR for *CXCL10* normalized to *TBP* as housekeeping gene (n=3 lines per condition for C9-healthy control (HC) pMGL and n=1 line per condition for IC-C9-2 pMGL comparison, n=3 differentiations each). **d)** ELISA for CXCL10 release into the culture supernatant (n=3 lines per condition for C9-HC pMGL and n=1 line per condition for IC-C9-2 pMGL comparison, n=3 differentiations each). **e)** ELISA

quantification showing no MMP9 release from C9 MNs (n=3 lines, n=1 differentiation each). **f)** ELISA quantification of MMP9 in supernatants after 48h-stimulation with TNF (50 ng/mL) and IL1B (20 ng/mL) (n=3 lines per condition, n=3-5 differentiations each for C9-HC comparison; n=1 line per condition, n=5 differentiations each for IC-C9-2 pMGL comparison). **g)** Left: Exemplar Western blot against pro- and active MMP9 normalized to the housekeeping gene b-actin after 48h-stimulation with TNF/IL1B. Right: Quantification showing significantly increased active MMP9 in C9 vs HC pMGL (n=3 lines per condition for C9-HC comparison, n=1 line for the IC-C9-2 comparison, n=4 differentiations each). **h)** Relative fold change for selected targets using a Protease Array on pMGL supernatant samples (pooled from n=3 lines per condition with n=2-3 differentiations each). **i)** Box plots of normalized counts from pMGL RNA-seq analysis for *CTSL*, *MMP1*, *MMP2*, *MMP7,* and *MMP8* (n=3 lines per condition). **j)** Relative fold change for selected targets using a Protease Inhibitor Array on pMGL supernatant samples (pooled from n=3 lines per condition with n=2-3 differentiations each). **k)** Box plots of normalized counts from pMGL RNA-seq analysis for *AGT* (Serpin A8*)*, *SERPINE1*, *TIMP1,* and (n=3 lines per condition). Single data points and means ± SEM or box plots. One-way ANOVA with Tukey's post-hoc test **(c/d)**. Two-tailed unpaired t-test (**f/g**). Source data are provided as a Source Data file.

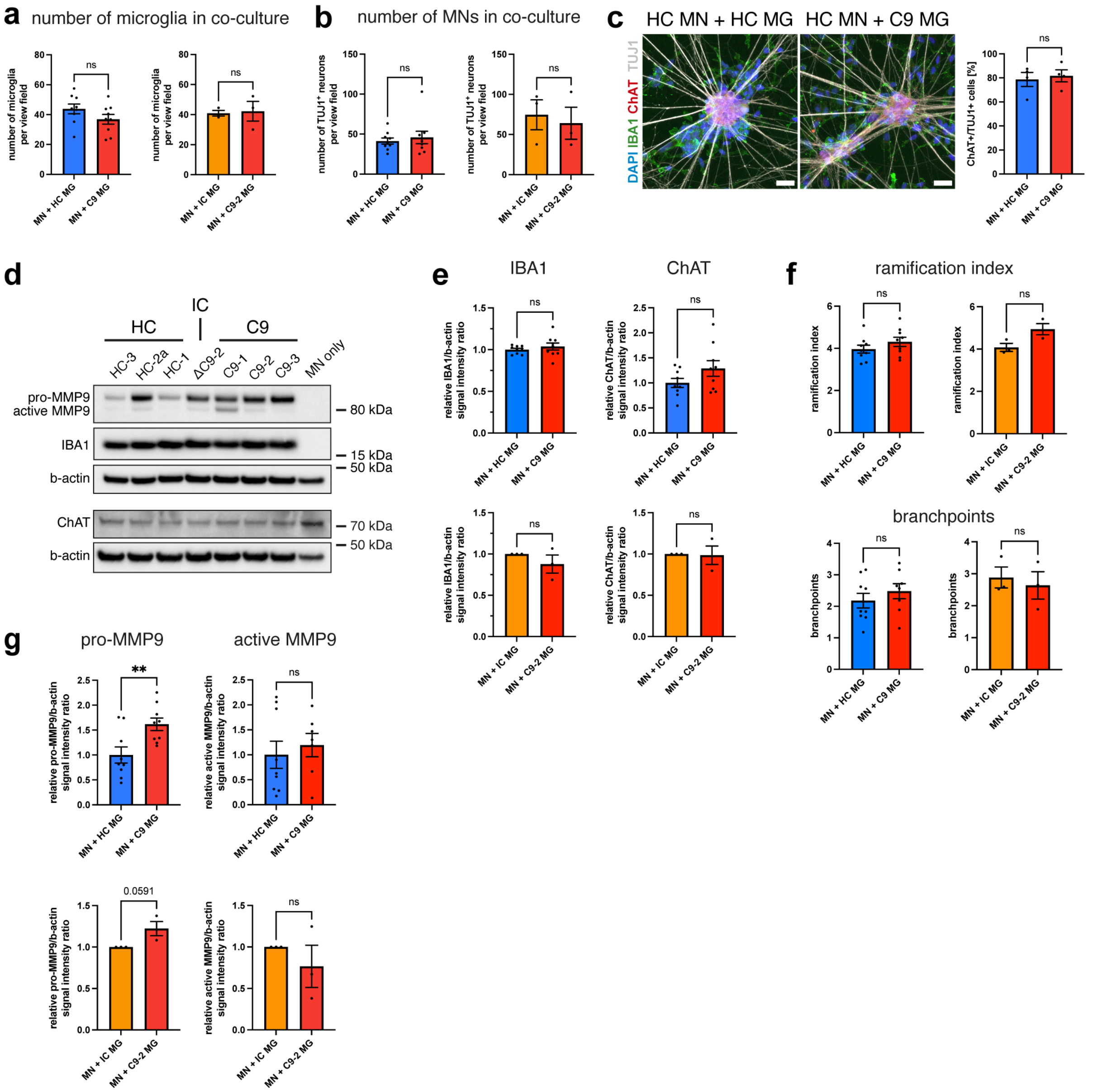

Supp. Fig. 6: Complementary analysis of unstimulated co-cultures of C9orf72 mutant and healthy iPSC microglia and healthy iPSC motor neurons. a/b) Quantification shows equal numbers of unstimulated healthy control (HC), isogenic control (IC), and C9orf72 mutant (C9) microglia (MG, **a**) in co-culture with healthy motor neurons (MNs, **b**) (n=3 microglial lines (C9-HC comparison) and n=1 line (IC-C9-2 comparison), n=3 differentiations each). **c)** Left: exemplar images showing expression of the MN marker ChAT in MNs in co-culture with HC and C9 MG. Right: Quantification showing equal ChAT expression in MNs in co-culture with HC and C9 MG (n=3 microglial lines, n=1-2 differentiations). Scale bars: 25 µm. **d)** Exemplar Western blot against IBA1, ChAT, and MMP9 in co-cultures of HC MNs with unstimulated HC, IC, and C9 microglia. **e)** Quantification of Western blot (in **d)** normalized to the housekeeping gene b-actin shows equal expression of the microglia marker IBA1 and the MN marker ChAT in co-cultures with unstimulated C9 and control MG (n=3 microglial lines for C9 and HC MG and n=1 microglial line for IC-C9-2 comparison, n=3 differentiations each). **f)** Quantification of microglial morphology using 3DMorph shows similar ramification index and number of branch points in C9 and control MG in co-culture with healthy MNs (n=3 microglial lines (C9-HC comparison) and n=1 microglial line (IC-C9-2 comparison), n=3 differentiations). **g)** Quantification of Western blot (in **d)** normalized to the housekeeping gene

b-actin shows significantly increased pro-MMP9 in the C9-HC comparison and a numeric increase in the IC-C9-2 comparison, while active MMP9 levels are unchanged (n=3 microglial lines for C9 and HC MG and n=1 microglial line for C9-2-IC comparison, n=3 differentiations each). Single data points and means ± SEM. Two-tailed unpaired t-test (**a-c, e-g**). Source data are provided as a Source Data file.

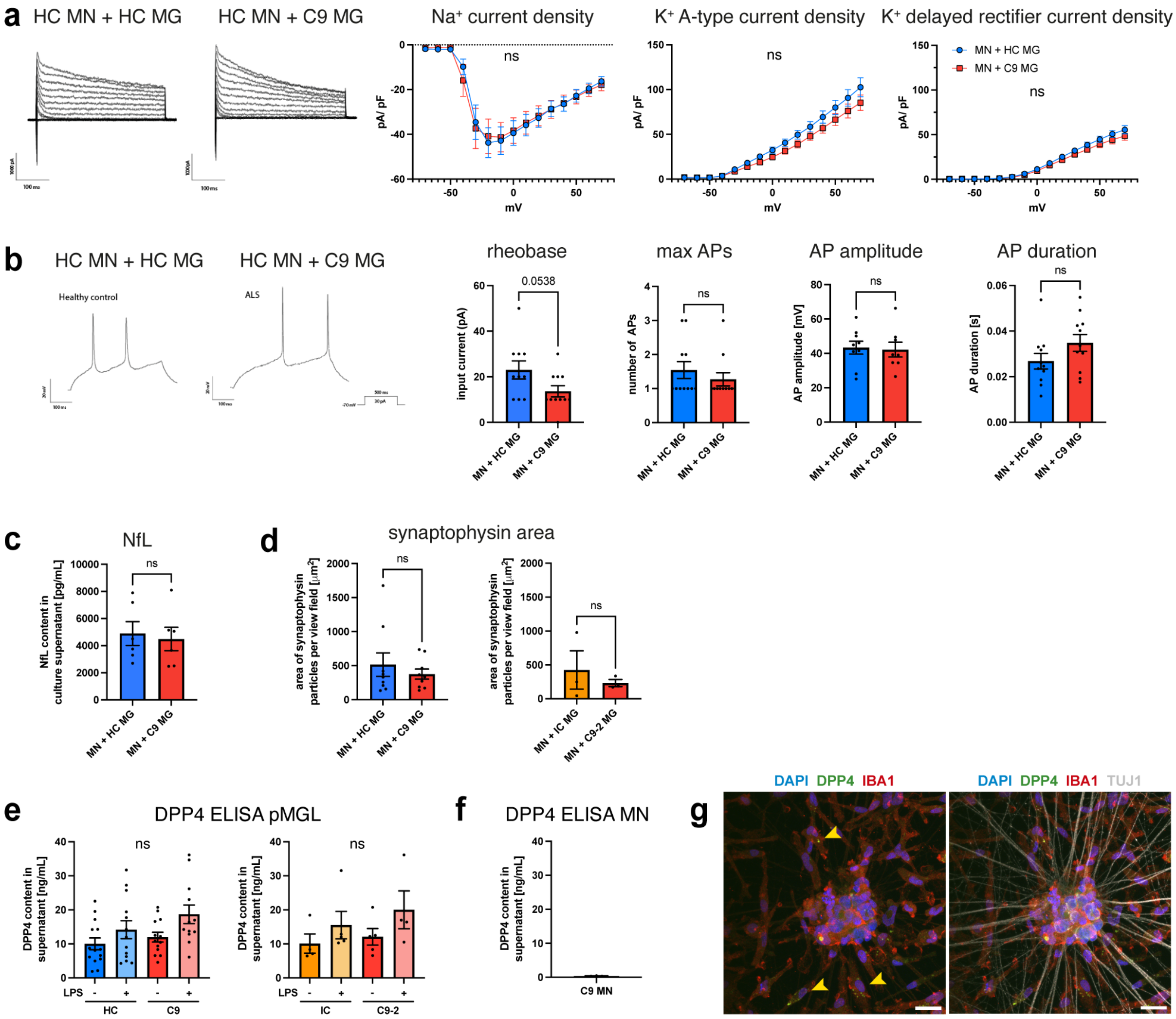

Supp. Fig. 7: Complementary analysis of unstimulated co-cultures of C9orf72 mutant and healthy iPSC microglia and healthy iPSC motor neurons. a) Left: Exemplar potassium and sodium currents in healthy control (HC) motor neurons (MNs) in co-culture with C9orf72 mutant (C9) and HC microglia (MG) using patch clamping. Right: Quantification shows no difference between HC MNs in co-culture with C9 and HC MG (co-cultures with n=3 microglial lines per condition with n=1 differentiations per line). **b)** Left: Exemplar induced action potentials (APs) in HC MNs in co-culture with C9 and HC MG using patch clamping. Right: Quantification of different AP parameters shows a trend to increased excitability but no statistical difference between HC MNs in co-culture with C9 and HC MG (single cells from co-cultures with n=3 microglial lines per condition with n=1 differentiations per line). **c)** Quantification of neurofilament light chain (NfL) release into the culture supernatant by MSD ELISA shows no difference between co-cultures with C9 and HC MG (co-cultures with n=3 microglial lines with n=2 differentiations each). **d)** Quantification of the area of synaptophysin particles shows no difference between co-cultures with C9 and control MG (n=3 microglial lines (C9-HC comparison) and n=1 microglial line (IC-C9-2 comparison), n=3 differentiations each). **e)** ELISA quantification shows no statistical difference in DPP4 content in culture supernatants from unstimulated and LPS-treated (100 ng/mL, 48 h) HC, IC, and C9 microglia in monoculture (pMGL) (n=3 lines per condition for C9-HC pMGL and n=1 line per condition for IC-C9-2 pMGL comparison, n=4-5 differentiations each). **f)** ELISA quantification shows DPP4 is barely released from C9 MNs in monoculture (n=3 lines, n=1 differentiation each). **g)** Exemplar immunofluorescent staining showing DPP4 expression co-localizes with IBA1-

positive cells, suggesting DPP4 is released by microglia in co-culture. Scale bars: 25 µm. Single data points and means ± SEM. Two-way ANOVA with Sidak's post-hoc test (**a**). Two-tailed unpaired t-test (**b-d**). One-way ANOVA with Tukey's post-hoc test (**e**). Source data are provided as a Source Data file.

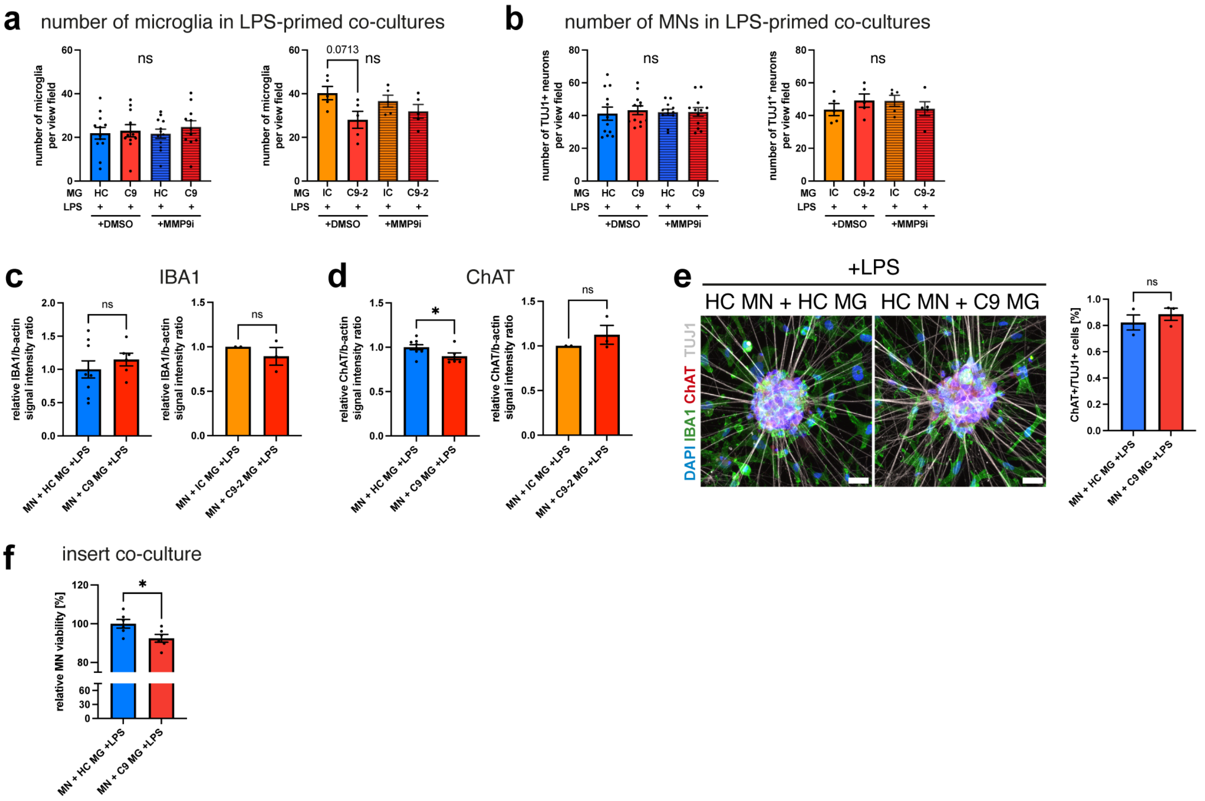

Supp. Fig. 8: Complementary analysis of LPS-stimulated co-cultures of C9orf72 mutant and healthy iPSC microglia with healthy iPSC motor neurons. a/b) Quantification showing equal numbers of healthy control (HC), isogenic control (IC), and C9orf72 mutant (C9) microglia (MG, **a**) in LPS-treated co-cultures with HC motor neurons (MNs, **b**) (co-cultures with n=3 microglial lines (C9-HC comparison) with n=3-4 differentiations each; n=1 microglial line (IC-C9-2 comparison) with n=5 differentiations each). **c/d)** Quantification of Western blot shown in **Fig. 5c** normalized to the housekeeping gene b-actin shows equal expression of the microglia marker IBA1 in LPS-treated co-cultures with C9 and control MG. The MN marker ChAT shows a mild but significant decrease in co-cultures with C9 compared with HC but not IC MG (co-cultures with n=2-3 microglial lines for C9 and HC MG and n=1 line for IC-C9-2 comparison, n=1-3 differentiations each). **e)** Left: exemplar images showing expression of the MN marker ChAT in MNs in LPS-treated co-cultures with HC and C9 MG. Right: Quantification showing equal ChAT expression in MNs in co-culture with HC and C9 MG (co-cultures with n=3 microglial lines, n=1 differentiation). Scale bars: 25 µm. **f)** MTS assay showing reduced MN viability in LPS-treated transwell co-cultures with C9 MG compared with HC MG (co-cultures with n=2 microglial lines, n=3 differentiations). Single data points and means ± SEM. One-way ANOVA with Tukey's post-hoc test (**a/b**) and two-tailed unpaired t-test (**c-f**). Source data are provided as a Source Data file.

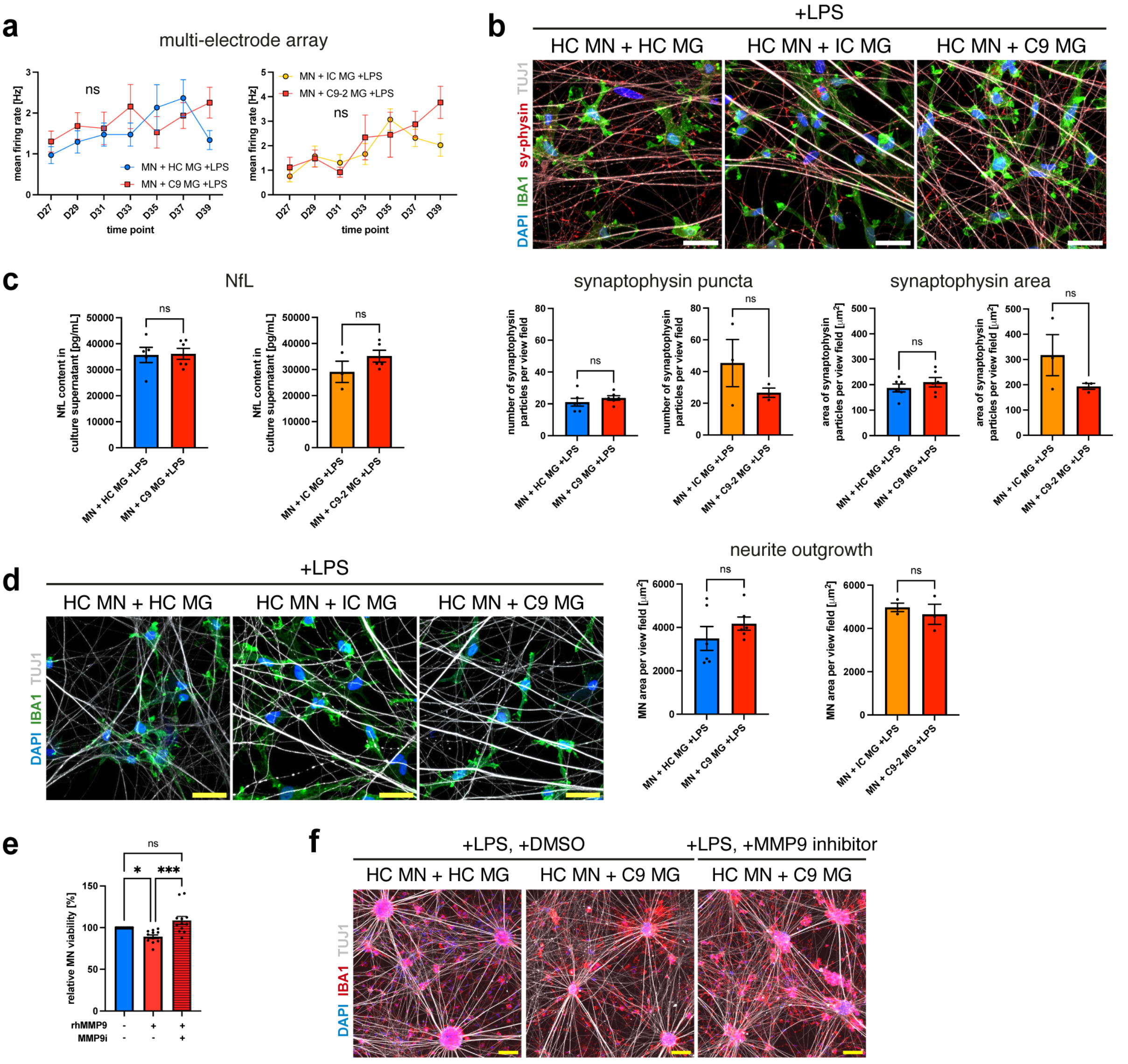

Supp. Fig. 9: Complementary analysis of LPS-stimulated co-cultures of C9orf72 mutant iPSC microglia with healthy iPSC motor neurons. a) Multi-electrode array analysis of healthy MNs in LPS-treated co-cultures with healthy control (HC), isogenic control (IC), and C9orf72 mutant (C9) microglia (MG) shows no differences in the mean firing rate (n=3 wells per differentiation for co-cultures with n=3 microglial lines (C9-HC comparison) and n=1 microglial line (IC-C9-2 comparison), n=2 differentiations each). **b)** Top: exemplar images of synaptophysin (sy-physin) staining in LPS-treated co-cultures with HC, IC, and C9 MG. Bottom: Quantification of the area and number of synaptophysin particles showing no difference between co-cultures with C9 and control MG (co-cultures with n=3 microglial lines (C9-HC comparison) and n=1 microglial line (IC-C9-2 comparison), n=2-3 differentiations each). Scale bars: 25 µm. **c)** Quantification of neurofilament light chain (NfL) release into the culture supernatant by MSD ELISA showing no difference between co-cultures with C9 and control MG (co-cultures with n=2 microglial lines (C9-HC comparison) and n=1 microglial line (IC-C9-2 comparison), n=2-5 differentiations each). **d)** Left: exemplar images of TUJ1-positive neurites in LPS-treated co-cultures with HC, IC, and C9 MG. Right: Quantification of neurite outgrowth showing no difference between co-cultures with C9 and control MG (co-cultures with n=3 microglial lines (C9-HC comparison) and n=1 microglial line (IC-C9-2 comparison), n=2-3 differentiations). Scale bars: 25 µm. **e)** MTS assay showing reduced viability of HC MNs after treatment with recombinant human MMP9 (rhMMP9, 30 ng/mL),

which is rescued by concomitant treatment with MMP9 inhibitor I (MMP9i, 3 µM) (n=3 lines, n=4 differentiations each). **f)** Same images as in **Fig. 6c** additionally showing the IBA1 staining in co-cultures of HC MNs with HC and C9 MG after pro-longed LPS treatment. Scale bars: 100 µm. Single data points and means ± SEM. Two-way ANOVA with Tukey's post-hoc test (**a**), two-tailed unpaired t-test (**b-d**), and one-way ANOVA with Tukey's post-hoc test (**e**). Source data are provided as a Source Data file.
