# Supplementary material for: C9orf72-ALS human iPSC microglia are pro-inflammatory and toxic to co-cultured motor neurons via MMP9
Source: Nat Commun. 2023 Sep 22;14:5898. doi: 10.1038/s41467-023-41603-0 (PMC10517114; doi:10.1038/s41467-023-41603-0)
Supplement: Supplementary file 3 — Reporting Summary [file 41467_2023_41603_MOESM3_ESM.pdf]

nature portfolio

Corresponding author(s): Kevin Talbot, Sally Cowley

Last updated by author(s): Aug 20, 2023

Reporting Summary

Nature Portfolio wishes to improve the reproducibility of the work that we publish. This form provides structure for consistency and transparency in reporting. For further information on Nature Portfolio policies, see our Editorial Policies and the Editorial Policy Checklist.

Statistics

For all statistical analyses, confirm that the following items are present in the figure legend, table legend, main text, or Methods section.

n/a	Confirmed	
☐	☒	The exact sample size (*n*) for each experimental group/condition, given as a discrete number and unit of measurement
☐	☒	A statement on whether measurements were taken from distinct samples or whether the same sample was measured repeatedly
☐	☒	The statistical test(s) used AND whether they are one- or two-sided<br>*Only common tests should be described solely by name; describe more complex techniques in the Methods section.*
☒	☐	A description of all covariates tested
☐	☒	A description of any assumptions or corrections, such as tests of normality and adjustment for multiple comparisons
☐	☒	A full description of the statistical parameters including central tendency (e.g. means) or other basic estimates (e.g. regression coefficient) AND variation (e.g. standard deviation) or associated estimates of uncertainty (e.g. confidence intervals)
☐	☒	For null hypothesis testing, the test statistic (e.g. *F*, *t*, *r*) with confidence intervals, effect sizes, degrees of freedom and *P* value noted<br>*Give P values as exact values whenever suitable.*
☒	☐	For Bayesian analysis, information on the choice of priors and Markov chain Monte Carlo settings
☒	☐	For hierarchical and complex designs, identification of the appropriate level for tests and full reporting of outcomes
☒	☐	Estimates of effect sizes (e.g. Cohen's *d*, Pearson's *r*), indicating how they were calculated

Our web collection on statistics for biologists contains articles on many of the points above.

Software and code

Policy information about availability of computer code

Data collection	AXIS software v2.5.2 (Axion BioSystems), Clampfit 10.3 (pCLAMP Software suite, Molecular Devices)
Data analysis	GraphPad Prism 9 (GraphPad Software), Fiji v1.53q (Schindelin et al., 2012), 3DMorph (York et al., 2018), ImageStudioLite v5.2.5 (LI-COR), Matlab R2022a (MathWorks), RStudio 1.4.1103 with HISAT2 v2.2.1, FeatureCounts v2.0.1, DESeq2 v1.28.1 and clusterProfiler v3.16.1, Adobe Illustrator 25.4.1

For manuscripts utilizing custom algorithms or software that are central to the research but not yet described in published literature, software must be made available to editors and reviewers. We strongly encourage code deposition in a community repository (e.g. GitHub). See the Nature Portfolio guidelines for submitting code & software for further information.

Data

Policy information about availability of data

All manuscripts must include a data availability statement. This statement should provide the following information, where applicable:

- Accession codes, unique identifiers, or web links for publicly available datasets
- A description of any restrictions on data availability
- For clinical datasets or third party data, please ensure that the statement adheres to our policy

All data supporting the findings of this study are available within the paper and its Supplementary Information. Source data are provided with this paper. The RNA

seq data generated in this study have been deposited in the GEO repository under accession code GSE217625 [https://www.ncbi.nlm.nih.gov/geo/query/acc.cgi?&acc=GSE217625]. Paired-end reads were mapped to the human GRCh38.p13 reference genome (https://www.gencodegenes.org).

Research involving human participants, their data, or biological material

Policy information about studies with human participants or human data. See also policy information about sex, gender (identity/presentation), and sexual orientation and race, ethnicity and racism.

Reporting on sex and gender	We used a matched approach (2 male lines, 1 female line for both C9-ALS patient and control lines to generate microglia). Information on the sex of the iPSC lines is provided below. These numbers do not allow for a meaningful sex-disaggregated analysis, therefore no sex-disaggregated analyses were performed.
Reporting on race, ethnicity, or other socially relevant groupings	n/a
Population characteristics	Information on the age of the iPSC lines is provided below.
Recruitment	n/a
Ethics oversight	All iPSC lines were previously derived from skin biopsy fibroblasts, collected under ethical approval granted by the South Wales Research Ethics Committee (WA/12/0186) and the South Central Berkshire Research Ethics Committee (REC10/H0505/71) in the James Martin Stem Cell Facility, University of Oxford, under standardized protocols

Note that full information on the approval of the study protocol must also be provided in the manuscript.

Field-specific reporting

Please select the one below that is the best fit for your research. If you are not sure, read the appropriate sections before making your selection.

☒ Life sciences ☐ Behavioural & social sciences ☐ Ecological, evolutionary & environmental sciences

For a reference copy of the document with all sections, see nature.com/documents/nr-reporting-summary-flat.pdf

Life sciences study design

All studies must disclose on these points even when the disclosure is negative.

Sample size	No sample-size calculation was performed; the number of different lines/differentiations used in the study represents common practice in the field (e.g., our previous publications: Vahsen et al., 2022, Sci Rep; Dafinca et al., 2016, Stem Cells; Dafinca et al., 2020, Stem Cell Rep). iPSC lines from n=3 different healthy individuals and n=3 different ALS patients were used to ensure reproducibility of experimental data across different individuals.
Data exclusions	No data were excluded from the analyses.
Replication	Multiple independent differentiations per experiment (at least n=3) were performed to replicate data. All attempts at replication were successful.
Randomization	Cell culture wells were allocated to each group (treated, control, etc.) at random.
Blinding	Investigators were blinded during analyses if possible or analyses were performed using automated macros.

Reporting for specific materials, systems and methods

We require information from authors about some types of materials, experimental systems and methods used in many studies. Here, indicate whether each material, system or method listed is relevant to your study. If you are not sure if a list item applies to your research, read the appropriate section before selecting a response.

Materials & experimental systems

n/a	Involved in the study
☐	☒ Antibodies
☐	☒ Eukaryotic cell lines
☒	☐ Palaeontology and archaeology
☒	☐ Animals and other organisms
☒	☐ Clinical data
☒	☐ Dual use research of concern
☒	☐ Plants

Methods

n/a	Involved in the study
☒	☐ ChIP-seq
☒	☐ Flow cytometry
☒	☐ MRI-based neuroimaging

Antibodies

Antibodies used

cleaved caspase 3 rabbit 9661S Cell Signaling
IBA1 rabbit 019-19741 FUJIFILM Wako
IBA1 goat ab5076 Abcam
synaptophysin guineapig 101004 Synaptic Systems
TDP-43 mouse 60019-2-1g Proteintech
TMEM119 rabbit ab185337 Abcam
TUJ1 mouse 801201 Biolegend
b-actin mouse AM4302 ThermoFisher
C9orf72 mouse GTX634482 GeneTex
C9orf72 mouse GTX632041 GeneTex
ChAT goat ab144P Sigma-Aldrich
GAPDH mouse Nb300221 Novus Biologicals
MMP9 rabbit ab76003 Abcam
DPP4 rabbit 67138 Cell Signaling

Validation

cleaved caspase 3 rabbit 9661S Cell Signaling - detects human protein and suitable for ICC (https://www.cellsignal.com/products/primary-antibodies/cleaved-caspase-3-asp175-antibody/9661?site-search-type=Products&N=4294956287&Ntt=9661s&fromPage=plp&_requestid=1674644)
IBA1 rabbit 019-19741 FUJIFILM Wako - detects human protein and suitable for ICC (https://labchem-wako.fujifilm.com/us/product/detail/W01W0101-1974.html); our own data in the manuscript demonstrate that it is also suitable for WB, detecting a band of the correct size in microglial cultures but not in neurons
IBA1 goat ab5076 Abcam - detects human protein and suitable for ICC (https://www.abcam.com/iba1-antibody-ab5076.html)
synaptophysin guineapig 101004 Synaptic Systems - detects human protein and suitable for ICC (https://sysy.com/product/101004)
TDP-43 mouse 60019-2-1g Proteintech - detects human protein and suitable for ICC (https://www.ptglab.com/products/TARDBP-Antibody-60019-2-Ig.htm)
TMEM119 rabbit ab185337 Abcam - detects human protein and suitable for ICC (https://www.abcam.com/tmem119-antibody-microglial-marker-ab185337.html)
TUJ1 mouse 801201 Biolegend - detects human protein and suitable for ICC (https://www.biolegend.com/en-us/products/purified-anti-tubulin-beta-3-tubb3-antibody-11580?GroupID=GROUP686)
b-actin mouse AM4302 ThermoFisher -
detects human protein and suitable for WB (https://www.thermofisher.com/antibody/product/beta-Actin-Antibody-clone-AC-15-Monoclonal/AM4302)
C9orf72 mouse GTX634482 GeneTex - detects human protein and suitable for WB (https://www.genetex.com/Product/Detail/C9orf72-antibody-GT1553/GTX634482)
C9orf72 mouse GTX632041 GeneTex - detects human protein and suitable for WB (https://www.genetex.com/Product/Detail/C9orf72-antibody-GT779/GTX632041)
ChAT goat ab144P Sigma-Aldrich - detects human protein and suitable for ICC and WB (https://www.merckmillipore.com/GB/en/product/Anti-Choline-Acetyltransferase-Antibody,MM_NF-AB144P)
GAPDH mouse Nb300221 Novus Biologicals - detects human protein and suitable for WB (https://www.novusbio.com/products/gapdh-antibody-1d4_nb300-221)
MMP9 rabbit ab76003 Abcam - detects human protein and suitable for WB (https://www.abcam.com/mmp9-antibody-ep1254-ab76003.html)
DPP4 rabbit 67138 Cell Signaling - detects human protein and suitable for ICC (https://www.cellsignal.com/products/primary-antibodies/dpp4-cd26-d6d8k-rabbit-mab/67138)

Eukaryotic cell lines

Policy information about cell lines and Sex and Gender in Research

Cell line source(s)

All iPSC lines were previously derived from skin biopsy fibroblasts in the James Martin Stem Cell Facility, University of Oxford, under standardized protocols.
OX3-06 male 49 years - PMID 29604226
SFC856-03-04 female 78 - 28827786
SFC840-03-03 female 67 - 26905200

	SFC841-03-01 male 36 - 27097283<br>C9-01-07 male 72 32330447<br>C9-02-02 female 58 27097283<br>C9-04-12 male 39 32330447<br>59-2 female 58 32504093
Authentication	All iPSC lines were generated in-house and have been published and characterized extensively before (see the manuscript for details). Cells lines were not re-authenticated for this study.
Mycoplasma contamination	All cell lines tested negative for mycoplasma contamination.
Commonly misidentified lines<br>(See ICLAC register)	No commonly misidentified cell lines were used in the study.
